# ﻿*Pternopetalum wulingense* (Apiaceae), a new species from the Wuling Mountains, China

**DOI:** 10.3897/phytokeys.263.163711

**Published:** 2025-09-24

**Authors:** Xuyang Yue, Xiaotian Pan, Ying Liu, Jianfei Ye

**Affiliations:** 1 State Key Laboratory of Biocontrol, School of Ecology, Shenzhen Campus of Sun Yat-sen University, No. 66, Gongchang Road, Guangming District, Shenzhen, 518107, Guangdong, China Sun Yat-sen University Shenzhen China

**Keywords:** Apiaceae, phylogeny, *
Pternopetalum
*, taxonomy

## Abstract

*Pternopetalum wulingense* (Apiaceae) is described as a new species from the Wuling Mountains in Guizhou Province and Chongqing Municipality, China. Both phylogenetic analyses and diagnostic morphological traits support its placement within *Pternopetalum* and confirm its distinction from close relatives. It closely resembles *P. trichomanifolium* in having finely dissected 3–4-pinnate basal leaves and a preference for moist habitats, but differs in having a rhombic leaf blade outline, ultimate segments of uniform width and oblong-ovoid fruits with both mericarps fully developed.

## ﻿Introduction

*Pternopetalum* Franch. (Apiaceae), a notable genus within tribe Apieae of subfamily Apioideae, comprises approximately 20 species and is endemic to East Asia (Wang 2012). It is amongst the largest genera of Apiaceae in this region (Pimenov and Leonov 2004). The genus is distributed across South Korea, Japan, China and adjacent eastern Himalayan regions (Pu 1985; Pimenov and Leonov 1993; Pu and Phillippe 2005), with a diversity centre in the East Himalaya–Hengduan Mountains region (Su and Sheh 2001). *Pternopetalum* is morphologically characterised by petals saccate at the base, umbellules with only 2–4 (–5) flowers and reflexed styles and rays in fruit (Pu 1985; Pu and Phillippe 2005; Wang 2012). After Wang’s (2012) comprehensive revision of the genus, five additional species have been described (Bhaumik and Satyanarayana 2014; Tan et al. 2014, 2015; Zhong et al. 2018; Ye et al. 2020) and, more recently, *Pternopetalum shunhuangensis* was described from Hunan Province by Zhou et al. (2025).

During a botanical survey in May 2015 in Mount Fanjing, Yinjiang County, Guizhou Province, China, we encountered an unusual population of *Pternopetalum*. Although it resembled *P. trichomanifolium* as described in Flora Reipublicae Popularis Sinicae (Pu 1985), it differed by having a rhombic leaf blade outline, ultimate segments of uniform width and oblong-ovoid fruits with both mericarps fully developed. To verify its taxonomic status, we revisited the site in October 2019, May 2024 and May 2025 to collected additional specimens with fruits. Furthermore, populations of the same taxon were subsequently documented in the adjacent Xiushan County, Chongqing Municipality, which borders Yinjiang County.

To test the generic affiliation of the unknown plant and clarify its relationship with its closest relatives within the genus, we conducted phylogenetic analyses, based on chloroplast DNA sequence data, including 16 currently recognised species of *Pternopetalum*. The results confirmed our hypothesis that this plant represents a previously undescribed species, which we formally describe here as *Pternopetalum wulingense*.

## ﻿Materials and methods

### ﻿Morphological observation

Specimens were collected in October 2019, May 2024 and May 2025 from Yinjiang County, Guizhou Province. In addition, specimens in April 2024 from Xiushan County, Chongqing Municipality, were also examined in this study. Habitat characteristics of the new species were documented through field investigations. The species was also cultivated in the greenhouse of China National Botanical Garden, Institute of Botany, Chinese Academy of Sciences, where additional morphological traits, including flower colour, were observed during the flowering period. Morphological descriptions were based on both dried specimens and cultivated plants.

### ﻿DNA extraction, amplification and sequencing

To test the generic affiliation of *Pternopetalum wulingense* and determine its phylogenetic position within the genus, we assembled a dataset comprising three chloroplast markers (*rpl16*, *rps16*, *trnL–trnF*) and the nuclear ribosomal internal transcribed spacer (nrITS). In total, 16 currently recognised species of *Pternopetalum* were included as ingroup taxa, with two additional species selected as outgroups. Sequences of *P. wulingense* were newly generated in this study, while the remaining sequences were retrieved from GenBank. Detailed information on the material sources and GenBank accession numbers is provided in Suppl. material 1.

Total genomic DNA of *P. wulingense* was extracted from fresh leaf tissue using a modified CTAB protocol (Doyle and Doyle 1987). The nrITS region was assembled using GetOrganelle v.1.7.7.1 (Jin et al. 2020). Chloroplast genomes were assembled using NOVOPlasty v.4.3.5 (Dierckxsens et al. 2017), with the built-in Seed_RUBP_cp.fasta as the seed sequence. The assembled plastomes were annotated using GeSeq (https://chlorobox.mpimp-golm.mpg.de/geseq.html) and the three chloroplast DNA sequences used in the phylogenetic analysis were extracted. These sequences (*rpl16*, *rps16*, *trnL–trnF* and nrITS) were then aligned with downloaded sequences of other species using MAFFT v.7.526 (Katoh et al. 2002) with default parameters and subsequently manually adjusted using MEGA6 (Tamura et al. 2013).

The supermatrix dataset for phylogenetic analysis was generated by concatenating three chloroplast markers and the nrITS sequence using BioEdit v.7.0.9.0 (Hall 1999). IQ-TREE 2 (Bui et al. 2020) was used to select the best-fit nucleotide substitution model for four DNA sequence partitions and to construct a Maximum Likelihood (ML) tree with 1000 bootstrap replicates. In the ML analysis, bootstrap support (BS) values < 50% were considered unreliable, values between 50–75% moderately supported and values > 75% strongly supported.

For Bayesian Inference (BI), PartitionFinder2 (Lanfear et al. 2016) was employed to determine the optimal substitution models for four DNA sequence partitions. BI analyses were conducted using MrBayes v.3.2.7 (Ronquist et al. 2012) under the selected model with the following settings: mcmc ngen = 2,000,000; nruns = 2; nchains = 4; samplefreq = 1000; temp = 0.2; sumt burnin = 2000. In the BI analysis, posterior probability (PP) values < 0.90 were considered poorly supported, values between 0.90–0.95 moderately supported and values > 0.95 strongly supported.

## ﻿Results and discussion

**Figure 1.**
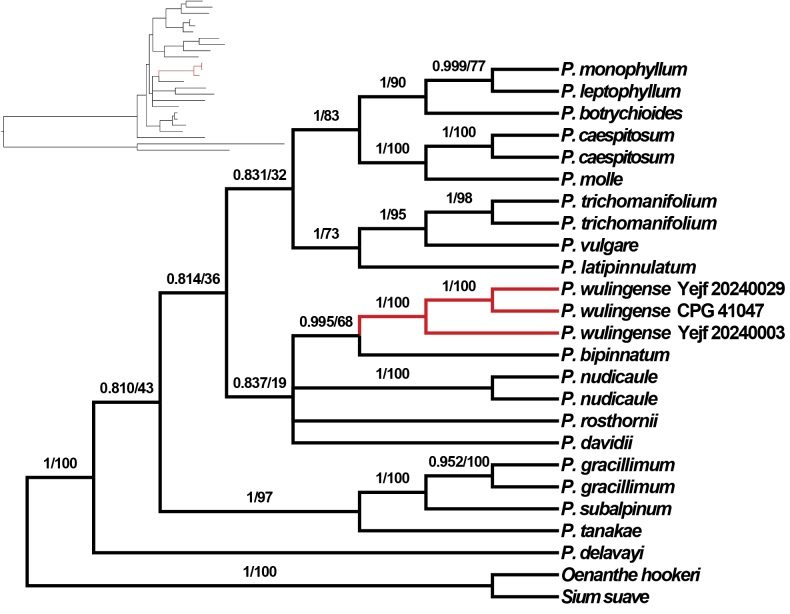
Phylogenetic tree inferred from the supermatrix dataset (including three chloroplast markers and the nrITS sequence). Bayesian posterior probability values (PP) / Bootstrap support values (BS) are shown on the branches. Only branches with PP > 0.5 are shown.

The phylogenetic tree inferred from the supermatrix dataset (Fig. 1) shows that *Pternopetalum wulingense* is sister to *P. bipinnatum* Li Song Wang with strong to moderate support (PP = 0.995; BS = 68%). Although *P. wulingense* is morphologically similar to *P. trichomanifolium* (Franch.) Hand.-Mazz, possibly due to similar moist habitat pressures, the two are genetically distant.

**Figure 2.**
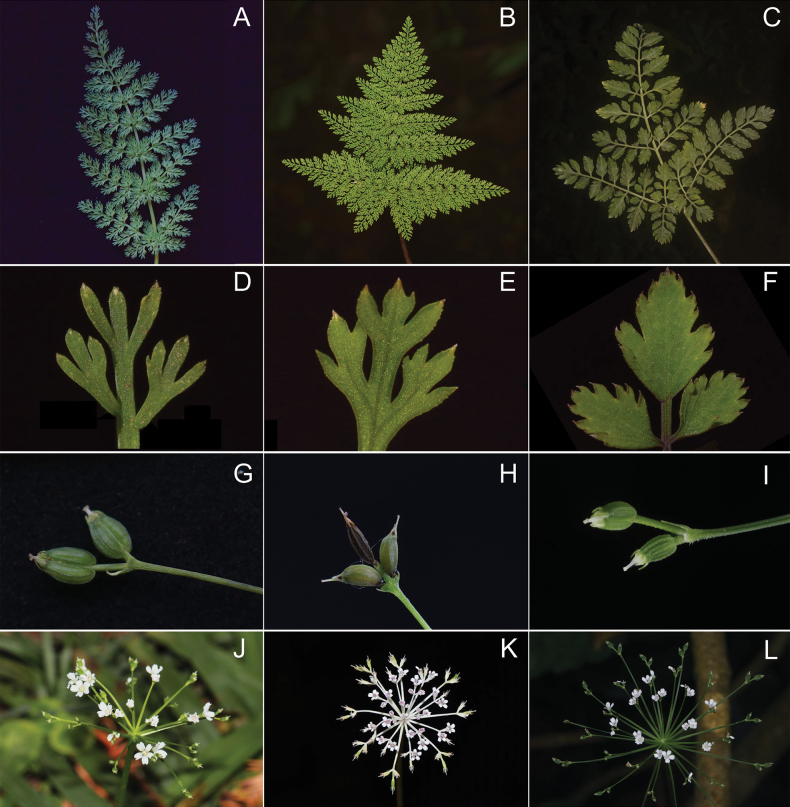
Morphological comparison of *Pternopetalum wulingense* (A, D, G, J), *P. trichomanifolium* (B, E, H, K) and *P. bipinnatum* (C, F, I, L). A–C. Leaf blade; D–F. Ultimate segments; G–I. Fruit; J–L. Inflorescence. Photograph credits: A, D by Xuyang Yue; B, C, E, H by Jianfei Ye; F, I by Xinjie Zhao; G by Kai Xue; H by Xinxin Zhu.

Upon examination of herbarium specimens, we discovered that an additional collection of this species had been made in 1979 from Xiushan County of Chongqing Municipality, but it was misidentified as *P. trichomanifolium*. Morphologically, the new species resembles *P. trichomanifolium* in having finely dissected 3–4-pinnate leaves and white obovate petals with incurved apices, but it differs in having a rhombic leaf blade outline, ultimate segments of uniform width and oblong-ovoid fruits with both mericarps fully developed (Table 1, Fig. 2). *P. wulingense* and *P. bipinnatum* share several similarities: both have two developed mericarps with prominent and glabrous ribs. Nevertheless, they exhibit distinct differences: *P. wulingense* is characterised by 3–4-pinnate leaves with linear ultimate segments, whereas *P. bipinnatum* has 2-pinnate leaves with oblong ultimate segments.

**Table 1.** Comparision of *Pternopetalum wulingense* sp. nov. and morphologically similar species. Morphological characters obtained from Wang (2005) and our field observations.

Character	*P. wulingense*	*P. trichomanifolium*	*P. bipinnatum*
Plant (cm)	10–30	30–40	10–30
Root	fibrous	fibrous	fibrous
Stem	1–4	1–3	2–3
Leaves	basal, 3–4-pinnate, very finely dissected, petiole 3–16 cm long	basal, nearly ternate-3–4-pinnate, very finely dissected, petiole 3–19 cm long	basal, 2-pinnate, petiole 3–9 cm long
Leaflets	ultimate segments linear, of uniform width, 1–4 mm long, < 1 mm broad, membraneous	ultimate segments linear, of variable width, 1.5–4 mm long, < 1 mm broad, membraneous	ultimate segments oblong, 0.5–1.5 cm long, 0.5–1 cm broad, at the margin thin coriaceous, mucronate-crenate and thickened at the border
Rays	12–26, 1–3 cm long	15–30, 2–5 cm long	15–22, 3–4 cm long
Umbellules	2–3-flowered, white anther	3–4-flowered, purple anther	2–3-flowered, light purple anther
Bracteoles	2, subulate	2–4, lanceolate	2, linear
Calyx teeth	triangular	subulate	triangular
Petals	white, obovate, with incurved apex	white, obovate, with incurved apex	white, oblong, without incurved apex
Styles	slightly recurved	erected	slightly recurved
Fruits	oblong-ovoid, ca. 1.5–3 mm long, 1.5–2 mm in diam., two mericarps are developed, ribs prominent and glabrous	narrow elliptical, ca. 3–4 mm long, 1 mm in diameter, often only one mericarp developed in fruit, ribs filiform	round, ca. 2–2.5 mm long, 1.5–2 mm in diam., two mericarps are developed in fruit, ribs prominent and glabrous
Vittae	1–2	1–3	1–2

### ﻿Taxonomic treatment

#### ﻿ Pternopetalum wulingense

Taxon classificationPlantaeApialesApiaceae

J.F.Ye, X.Y.Yue & Ying Liu sp. nov.

2FF56DBD-4E7B-5040-945B-2AD5D249B09F

urn:lsid:ipni.org:names:77369654-1

Figs 3, 4

##### Type.

China • Guizhou Province, Yinjiang County, Fanjingshan Mountain (27.92°N, 108.65°E), on moist rock walls by the stream, at an altitude of ca. 1300 m a.s.l., 01 May 2025, in fruit, *X. Y. Yue & B. K. Yang Yejf20250078* (holotype: SYS!; isotypes: SYS!, PE!).

***Paratype***: China • Chongqing Municipality, Xiushan County, Aikou Town (28.27°N, 108.83°E), under forest, at an altitude of ca. 1100 m a.s.l., 18 April 2024, in fruit, *S. R. Yi Yejf20240003* (paratype: SYS!). • China. Rongxi Town, Xiushan County, Chongqing Municipality, damp shade, 24 June 1979, *Anonymous 0785* (SM).

##### Etymology.

The specific epithet *wulingense* refers to the Wuling Mountains (武陵山), where the new species was discovered.

##### Diagnosis.

**Figure 3.**
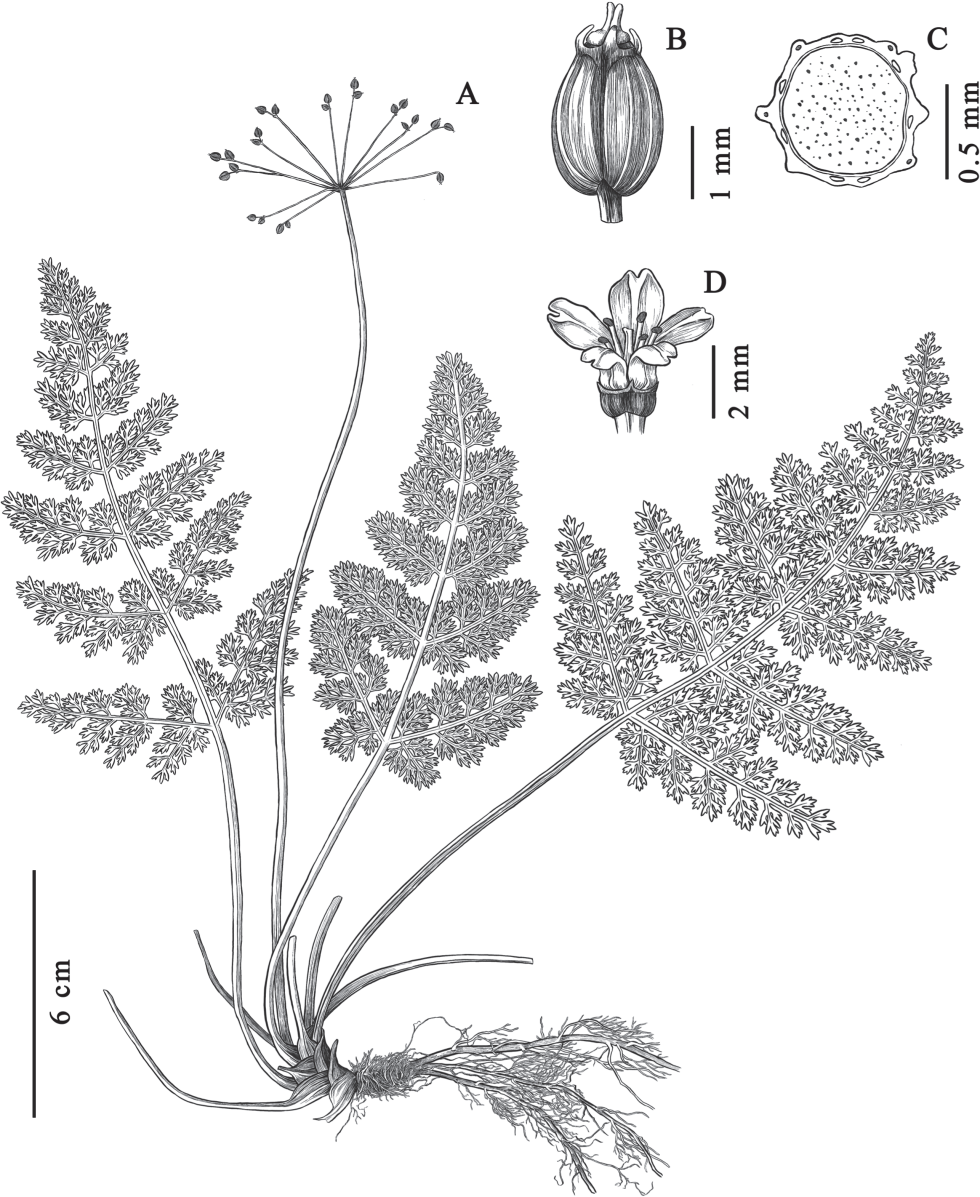
*Pternopetalum wulingense* J.F.Ye, X.Y.Yue & Ying Liu. A. Habit; B. Fruit; C. Transaction of mature fruit; D. Flower. Drawn by Xiaotian Pan.

**Figure 4.**
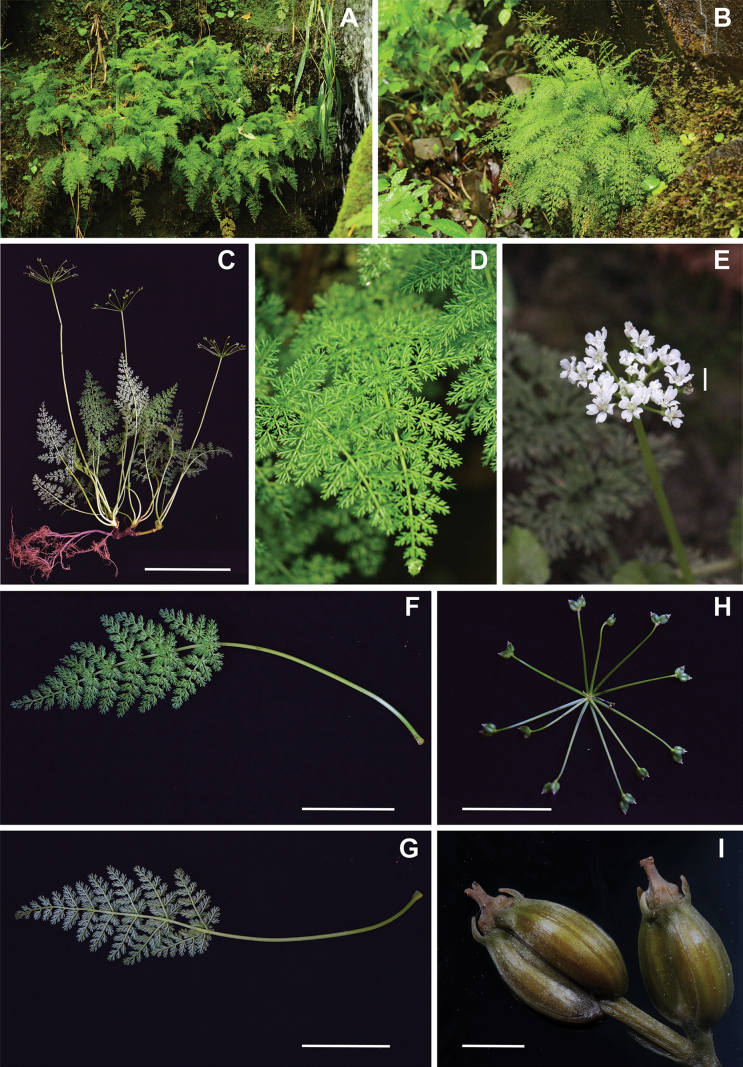
*Pternopetalum wulingense* J.F.Ye, X.Y.Yue & Ying Liu. A. Microhabitat; B,C. Habit; D. Basal leaf blade; E. Flowers; F. Basal leaf blade adaxially; G. Basal leaf blade abaxially; H. Infructescence; I. Umbellule and fruits. Scale bars: 10 cm (C, F, G); 5 mm (E); 5 cm (H); 1 mm (I). Photograph credits: A, B, D by Jianfei Ye; C, F, G, H, I by Xuyang Yue; E by Kai Xue.

*Pternopetalum wulingense* differs from *P. trichomanifolium* by having a rhombic leaf blade outline, ultimate segments of uniform width and oblong-ovoid fruits with both mericarps fully developed (Table 1, Figs 2–4).

##### Description.

Plants 10–30 cm tall. Stems 1–4, erect. Leaves mostly basal, petiolate; petioles 3–16 cm; blades triangular-ovate in outline, 3–9 × 4–13 cm, 3–4-pinnate, finely dissected; ultimate segments linear, 1–4 × 0.2–0.5 mm, membranous. Bracts absent. Rays 12–26, 1–3 cm. Bracteoles 2, subulate, 0.5–1.5 mm. Umbellules 2–3-flowered; pedicels 0.2–1.5 mm. Calyx teeth triangular. Petals white, obovate, with incurved apices. Stylopodium conical; styles slightly recurved. Fruits oblong-ovoid, 1.5–3 × 1.5–2 mm, with both mericarps developed; ribs prominent and glabrous; vittae 1–2 per furrow.

##### Phenology.

*Pternopetalum wulingense* flowering from March to April and fruiting from May to June.

##### Distribution and habitat.

*Pternopetalum wulingense* is currently known only from Yinjiang County, Guizhou Province and adjacent Xiushan County, Chongqing Municipality, China. It grows on moist rock walls along streams at around 1300 m a.s.l., often co-occurring with mosses. Associated species include *Cardamine* sp., *Dichocarpum* sp., *Elatostema* sp., *Impatiens* sp., *Pilea* sp., *Saxifraga* sp. and *Sedum* sp.

##### Vernacular name.

武陵囊瓣芹 [wŭ líng náng bàn qín].

##### Conservation status.

As no comprehensive population assessment has yet been conducted across its known range, we recommend assigning the new species a conservation status of Data Deficient (DD) under the IUCN Red List Criteria (IUCN 2017). Further field surveys are needed to assess its population size, range and potential threats.

## Supplementary Material

XML Treatment for Pternopetalum wulingense

## ﻿Additional information

### Use of AI

AI tools were used only to polish the English language. No AI assistance was involved in data analysis, study design, or content generation.

### Author ORCIDs

Xuyang Yue https://orcid.org/0009-0004-4365-946X

Xiaotian Pan https://orcid.org/0009-0009-1653-5265

Ying Liu https://orcid.org/0000-0003-0613-837X

Jianfei Ye https://orcid.org/0000-0002-1869-4544

### Data availability

All of the data that support the findings of this study are available in the main text or Supplementary Information.

## Supplementary materials

Supplementary material 1 Source of materials studied and GenBank accession numbers Data type xls Explanation note Detailed information on the material sources and GenBank accession numbers. https://binary.pensoft.net/file/1423023This dataset is made available under the Open Database License (http://opendatacommons.org/licenses/odbl/1.0/). The Open Database License (ODbL) is a license agreement intended to allow users to freely share, modify, and use this Dataset while maintaining this same freedom for others, provided that the original source and author(s) are credited.Xuyang Yue, Xiaotian Pan, Ying Liu, Jianfei Ye
